# The Role of Oxidative Stress in the Aging Process

**DOI:** 10.1100/tsw.2010.94

**Published:** 2010-06-15

**Authors:** Barbara F. Oliveira, José Augusto Nogueira-Machado, Míriam M. Chaves

**Affiliations:** ^1^ Biochemistry of Aging and Correlated Diseases, Biochemistry and Immunology Department, Biological Sciences Institute, Federal University of Minas Gerais, BH/MG, Brazil; ^2^ Santa Casa Hospital of Belo Horizonte, MG, Brazil

**Keywords:** oxidative stress, aging, immune system, free radicals

## Abstract

The aging of organisms is characterized by a gradual functional decline of all organ systems. An appropriate theory must explain four main characteristics of aging: it is progressive, endogenous, irreversible, and deleterious for the individual. The aging of the immune system, or immunosenescence, is manifested by an increased susceptibility to infections with increased morbidity and mortality. Phagocytic capacity, synthesis of reactive oxygen intermediaries, and the intracellular killing efficiency of neutrophils are impaired in the elderly. Among all aging theories, the most updated one describes the free radicals. It implies that progressive aging is associated with higher levels of oxidative biomolecules reacted with free radicals. Although reactive oxygen species (ROS) are predominantly implicated in causing cell damage, they also play a major physiological role in several aspects of intracellular signaling and regulation. ROS include a number of chemically reactive molecules derived from oxygen. Not only oxygen, but also nitrogen can be deleterious species. The overproduction of reactive nitrogen species (RNS) is called nitrosative stress. ROS/RNS are known to play a dual role in biological systems since they can be either harmful or beneficial to living systems.

## AGING

Aging is a complex biological phenomenon often followed by various socioeconomic changes that have a great impact on the nutritional status and needs of the elderly individual. The incidence of disability increases with aging. Over one-third of elderly persons is limited by chronic conditions and is unable to carry on major activities[1].

The aging of organisms is characterized by a gradual functional decline of all organ systems. Whether cellular senescence contributes to an organism's aging has been controversial[2].

Part of the oddity about aging science thus derives from the fact that we must begin by dismantling important preconceptions about why aging occurs. Another part of the oddity about aging is the inherent complexity. Almost every aspect of an organism's phenotype undergoes modification with aging, and this phenomenological complexity has led, over the years, to a bewildering proliferation of ideas about specific cellular and molecular causes[3].

Why and how does aging occur? This is a complex scientific question that involves a large number of theories. However, an appropriate theory must explain four main characteristics of aging: it is progressive, endogenous, irreversible, and deleterious for the individual[4].

Fortunately, recent advances have resulted in significant simplification of the theoretical underpinnings of aging research and this, combined with the greatly increased power of experimental techniques to investigate the phenomenological complexities of the senescent phenotype, has helped to clear a path toward unraveling the workings of the aging process[3].

## AGING AND THE IMMUNE SYSTEM

The aging of the immune system, or immunosenescence, is manifested by an increased susceptibility to infections with increased morbidity and mortality. The immune system undergoes substantial change during aging. These changes are complex and include diminished strength of some parameters of immune response, qualitative alterations in response, and even exacerbation of some specific immune events. At present, the overall understanding of these age-related changes is very incomplete, but it is unlikely that all of these events reflect a single underlying molecular basis, or even a simple set of events. At this stage of incomplete and emerging understanding, it is therefore most judicious to pursue diverse research directions and strategies, including investigation of innate and adoptive immunity, attention to distinct cell types, and the use of cellular, genetic, and molecular tools to the best advantage[5].

All immune cells are affected by aging, contributing to the high susceptibility to infections and increased mortality observed in the elderly[6]. Evidence suggests that macrophages play a central role in both innate and adaptive immune responses. Intrinsic, as well as extrinsic (environmental), factors dictate macrophage function. In aged subjects, the influence of extrinsic factors becomes increasingly more important. This may override the innate immune balance, pro- vs. anti-inflammatory signals, thus yielding an inappropriate (either inadequate or overabundant) response when the system is challenged. Phagocytic capacity, synthesis of reactive oxygen intermediaries, and the intracellular killing efficiency of neutrophils are impaired in the elderly. For example, the production of nitrogen species is attenuated in macrophages from aged subjects relative to their younger counterparts[7].

Thus, there are differences in both the number and function of multiple cell types, which could have global effects on innate immunity[7].

Human granulocytes play a key role in host defenses against invading pathogens and are major effectors of the acute inflammatory reactions. In response to a variety of agents, neutrophils release large quantities of superoxide anion ($O_2^-$) in a phenomenon known as respiratory burst (Fig. 1). The inappropriate activation of respiratory burst is associated with tissue injury and impairment of the ability to clear invading microorganisms[8].

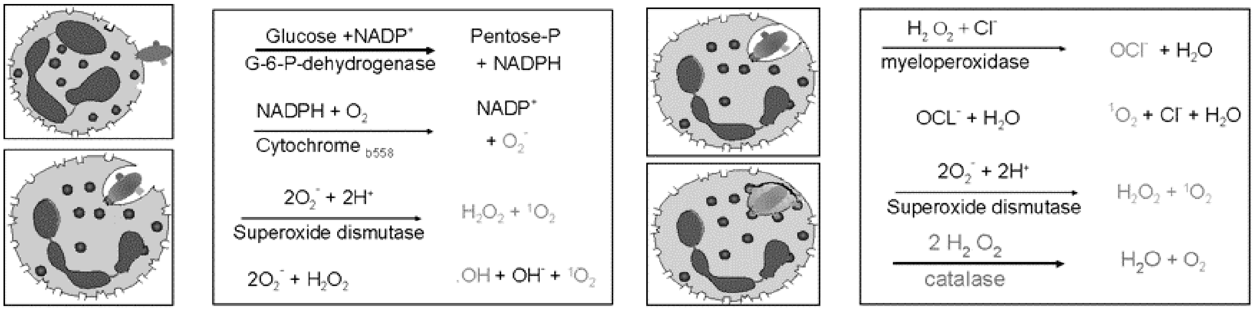

**FIGURE 1.** Reactive oxygen and nitrogen species generation through phagocyte respiratory burst. www.bdsfla.com

A strong parallelism between oxidizing species generation and cellular reducing power response in granulocytes has been reported. These results suggest a metabolic equilibrium and it has been suggested that the tissue damage occurs in a disequilibrium state between both responses[9].

## FREE RADICAL THEORY OF AGING

According to Medvedev[10], there are over 300 theories of aging, many of which agree among themselves by treating the same themes in different aspects. Many of the concepts described as aging theories are, thus, application of life science domains towards aging. Considering this context, in molecular genetics, proposals of altered proteins[11], somatic mutation[12], and telomer shortening[13] are discussed; in biochemistry, free radical theory[14] and metabolic rates[15]; and in evolution theories[16], those that emphasize aging not as a biological phenomenon in individuals, but as a process of evolution.

An important group of aging theories studies changes that occur and accumulate as time passes[17]. Among them, the most updated theory describes the free radicals. It implies that progressive aging is associated with higher levels of oxidative biomolecules reacted with free radicals. This theory has been continuously studied and modified during the years, and at the moment, free radicals are designated as intermediate oxygen species and intermediate nitrogen species that attack biomolecules. These intermediate species are products of normal cellular metabolism, considering that 1–2% of oxygen used in mitochondrial respiration is converted to reactive oxygen intermediates (ROI).

The causes of the poisonous properties of oxygen were obscure prior to the publication of Gerschman et al.'s free radical theory of oxygen toxicity in 1954, which states that the toxicity of oxygen is due to partially reduced forms of oxygen[18].

The world of free radicals in biological systems was soon thereafter explored by Harman, in 1956, who proposed the concept of free radicals playing a role in the aging process[19]. Elevations in the levels of oxidizing species generation from phagocytes without a concomitant increase in the reducing power was shown starting with the 40s age group, in spite of a significant decrease in the reducing power observed only starting from the 50s age group[20].

Intense research into the field of free radicals in biological systems has also been developing its relation to signaling processes for decades. Mittal and Murad provided evidence that the hydroxyl radical $^{\bullet}OH$ stimulates activation of guanylate cyclase and formation of the "second messenger" cyclic guanosine monophosphate (cGMP)[21].

The elderly present a lack of functional metabolic balance between cGMP and cAMP on ROI generation by neutrophils. There is an age-related exacerbation of the effect of cGMP on ROI generation, and a simultaneous lack of functional metabolic balance of cAMP and cGMP in subjects over 50 years of age. cGMP participates in inflammatory processes, while cAMP is important for regulating the anti-inflammatory process[22].

Signal transduction is a process that enables information to be transmitted from the outside of a cell to various functional elements inside the cell. Although reactive oxygen species (ROS) are predominantly implicated in causing cell damage, they also play a major physiological role in several aspects of intracellular signaling and regulation. It is a well-known feature that cells are capable of generating, endogenously and constitutively, ROS that are utilized in the induction and maintenance of signal transduction pathways involved in cell growth and differentiation[23].

Furthermore, correlation between age-related protein kinase C activity, NADPH oxidase phosphorylation, and ROS production have been suggested synergistically with unspecific and inflammatory responses, frequent in the elderly[24,25].

## REACTIVE OXYGEN SPECIES (ROS)

ROS have been emerging as critical signaling molecules. The term "reactive oxygen species" encompasses a wide range of molecules. Free radicals are chemical species containing one or more unpaired electrons. Examples include the hydrogen atom, with one unpaired electron, most transition metal ions, nitric oxide, and oxygen, which has two unpaired electrons. The unpaired electrons of oxygen react to form partially reduced, highly reactive species that are classified as ROS, including superoxide ($O_2^-$), hydrogen peroxide ($H_2O_2$), hydroxyl radical, and peroxynitrite. Various enzyme systems produce ROS, including the mitochondrial electron transport chain, cytochrome P450, lipoxygenase, cyclooxygenase, the NADPH oxidase complex, xanthine oxidase, and peroxisomes[26].

When phagocytes are activated, they produce ROS in amounts enough to kill intruding bacteria. In this system, ROS are produced by the NADPH oxidase complex that converts $O_2$ to $O_2^-$ (Fig. 2). Superoxide is then reduced in the phagosome by SOD to $H_2O_2$. That can be further converted to HOCl, which is highly toxic to bacteria and carries a direct antimicrobial effect of ROS.

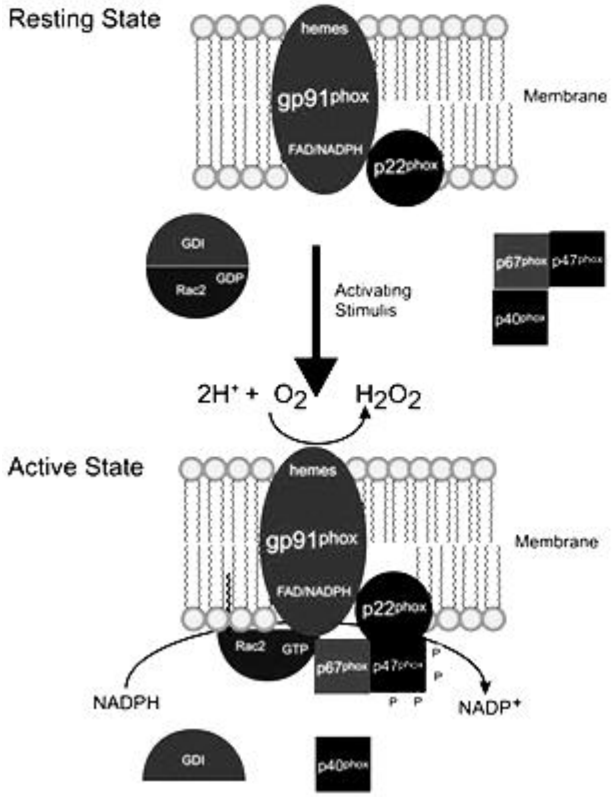

**FIGURE 2.** NADPH oxidase ROS production[31].

Even though the sources of ROS are numerous, including endogenous and exogenous sources, some advocates of the free radical theory of aging focus on ROS derived from the cellular metabolism taking place in mitochondria (Fig. 3). One hypothesis is that ROS production or leakage in mitochondria, rather than antioxidant defense systems, determines the rate of the aging process. Supporting this concept is a correlation between mitochondrial ROS production and longevity, observed at least for a few species[27].

ROS include a number of chemically reactive molecules derived from oxygen. Some of those molecules are extremely reactive, such as the hydroxyl radical, while some are less reactive (superoxide and hydrogen peroxide). Free radicals and ROS can readily react with most biomolecules, starting a chain

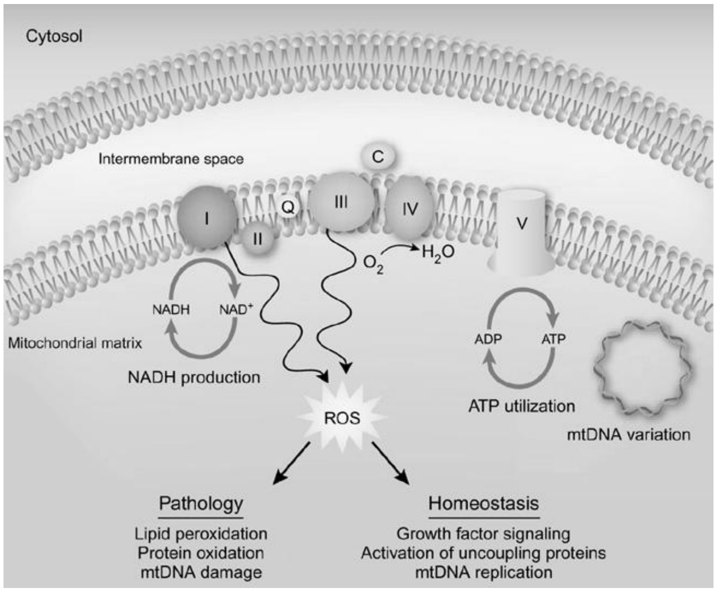

**FIGURE 3.** Mitochondrial electron transport chain ROS production[32].

reaction of free radical formation. In order to stop this chain reaction, a newly formed radical must either react with another free radical, eliminating the unpaired electrons, or react with a free radical scavenger — a chain-breaking or primary antioxidant[28].

In Table 1, the most common intracellular forms of ROS are listed, together with their main cellular sources of production and the relevant enzymatic antioxidant systems scavenging these ROS molecules[28].

**TABLE 1**
**Major ROS Molecules[28**

| ROS Molecule | Main Sources | Enzymatic Defense Systems | Product(s) |
|---|---|---|---|
| Superoxide ($O_2^-$) | "Leakage" of electrons from the electron transport chain | Superoxide dismutase (SOD) | $H_2O_2 + O_2$ |
| | | Superoxide reductase (in some bacteria) | $H_2O_2$ |
| | Activated phagocytes | | |
| | Xanthine oxidase | | |
| | Flavoenzymes | | |
| Hydrogen peroxide ($H_2O_2$) | From ($O_2^-$) via SOD | Glutathione peroxidase | $H_2O$ + GSSG |
| | | Catalases | $H_2O + O_2$ |
| | NADPH-oxidase (neutrophils) | Peroxiredoxins (Prx) | $H_2O$ |
| | Glucose oxidase | | |
| | Xanthine oxidase | | |
| Hydroxyl radical ($^{\bullet}OH$) | From $O_2^-$ and $H_2O_2$ via transition metals (Fe or Cu) | | |
| Nitric oxide (NO) | Nitric oxide synthases | Glutathione/TrxR | GSNO |

## REACTIVE NITROGEN SPECIES (RNS)

It has been almost 2 decades since the suggestion of the biological formation of peroxynitrite by the near diffusion-controlled reaction between nitric oxide (NO•) and superoxide ($O_2^-$) radicals and the implications for oxidative injury. This novel concept was built on some earlier chemical and physiological work that indicated the oxidative potential of peroxynitrite and the modulatory role of $O_2^-$ in the half-life of NO[29].

NO• is a small molecule that contains one unpaired electron on an antibonding orbital and is, therefore, a radical. NO• is generated in biological tissues by specific nitric oxide synthases (NOSs), which metabolize arginine to citrulline with the formation of NO• *via* a five-electron oxidative reaction. NO• is an abundant reactive radical that acts as an important oxidative biological signaling molecule in a large variety of diverse physiological processes, including neurotransmission, blood pressure regulation, defense mechanisms, smooth muscle relaxation, and immune regulation[23].

The field of research of peroxynitrite in RNS, with the recognition that not only peroxynitrite, but other related species derived from NO• redox metabolism participate in related reaction pathways, is broadening. Biologically generated RNS include the nitrogen dioxide radical (•$NO_2$). At present, the area of NO•-derived oxidants in biology represents a merging zone for NO• and redox metabolism, as well as strong implications in both cell signaling and oxidative damage[29].

The overproduction of RNS is called nitrosative stress. This may occur when the generation of RNS in a system exceeds the system’s ability to neutralize and eliminate them. Nitrosative stress may lead to nitrosylation reactions that can alter the structure of proteins and so inhibit their normal function[23].

NO potently increases oxygen supply to mammalian tissues by stimulating vascular relaxation. At higher concentrations, NO• inhibits oxygen consumption within tissues by inhibiting mitochondrial cytochrome oxidase, the main oxygen sink in tissues. Thus, because NO• stimulates oxygen supply while inhibiting oxygen consumption, NO• potentially increases oxygen level within mammalian tissues, which in turn may increase ROS production as the latter is often oxygen limited[30](Fig. 4).

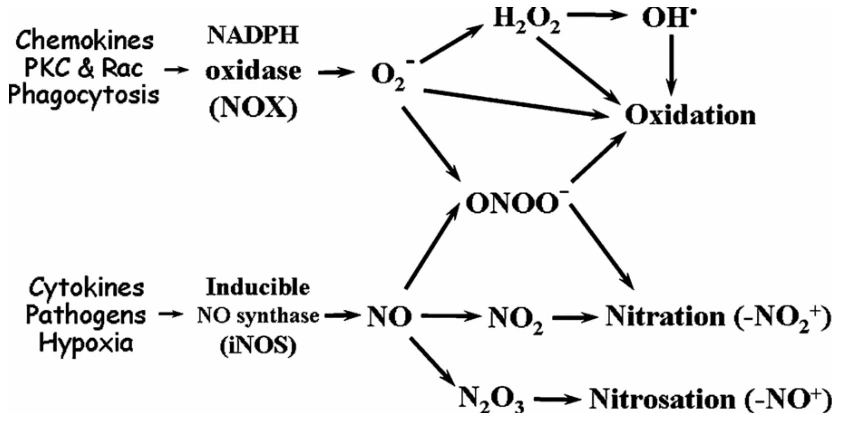

**FIGURE 4.** Generation and interaction of ROS and RNS[30].

## ROS AND RNS

ROS/RNS are known to play a dual role in biological systems since they can be either harmful or beneficial to living systems. Beneficial effects of ROS involve physiological roles in cellular responses to noxia, as, for example, in defense against infectious agents and in the function of a number of cellular signaling systems. One further beneficial example of ROS at low concentrations is the induction of a mitogenic response. In contrast, at high concentrations, ROS can be important mediators of damage to cell structures, including lipids and membranes, proteins, and nucleic acids (termed oxidative stress). The harmful effects of ROS are balanced by the antioxidant action of nonenzymatic antioxidants in addition to antioxidant enzymes. Despite the presence of the cell's antioxidant defense system to counteract oxidative damage from ROS, oxidative damage accumulates during the life cycle and radical-related damage to DNA, proteins, and lipids has been proposed to play a key role in the development of age-dependent diseases.

Therefore, we turn back to the issue once started to be discussed, the relation of oxidative stress with aging and all its consequences. More research must be done to clarify the exact mechanisms relating aging and oxidative stress, as well as the most wanted answer: how to postpone senescence or age with utmost health.
